# Sirtuin 7 Inhibitor Attenuates Colonic Mucosal Immune Activation in Mice—Potential Therapeutic Target in Inflammatory Bowel Disease

**DOI:** 10.3390/biomedicines10112693

**Published:** 2022-10-25

**Authors:** Sanghyun Kim, Junhyoung Byun, Semyung Jung, Byoungjae Kim, Kangwon Lee, Hanjo Jeon, Jaemin Lee, Hyuksoon Choi, Eunsun Kim, Yoontae Jeen, Hongsik Lee, Hoonjai Chun, Bora Keum, Taehoon Kim

**Affiliations:** 1 Department of Internal Medicine, College of Medicine, Korea University, Seoul 02841, Korea; 2 Department of Otorhinolaryngology-Head & Neck Surgery, College of Medicine, Korea University, Seoul 02841, Korea; 3 Mucosal Immunology Institute, College of Medicine, Korea University, Seoul 02841, Korea

**Keywords:** sirtuin, inflammatory bowel disease, SIRT7

## Abstract

Accumulating evidence has shown that sirtuin 7 (SIRT7), a mediator of various cellular activities, plays an important role in the pathogenesis of various immune-mediated inflammatory disorders. However, information remains limited regarding the role of SIRT7 in intestinal inflammation. We used a murine colitis model to investigate the role of SIRT7 in intestinal immunity and whether SIRT7 inhibitors could attenuate the intestinal inflammatory response. Mice were divided into three groups: control, colitis-induced, and SIRT7-inhibitor-treated. A colitis mouse model was established by intraperitoneal injection and nasal challenge with ovalbumin, as in our previous study. Quantitative analyses of inflammatory cytokines and SIRT7 levels in the colonic mucosa were performed to compare the changes in inflammatory responses between the three groups. The colitis group showed increased levels of inflammatory cytokines and SIRT7 in the colonic mucosa. The inflammatory reaction was suppressed in colitis-induced mice administered the SIRT7 inhibitor. The qRT-PCR results showed normalization of inflammatory cytokines in the SIRT7 inhibitor-treated group. Histologic study revealed a decrease in the extent of inflammation after SIRT7 treatment. We also observed that the degree of clinical inflammation was improved in SIRT7-treated mice. Our study demonstrated that SIRT7 inhibition attenuated the inflammatory response in the colon of mice, suggesting a possible role for SIRT7 in the pathogenesis of immune-mediated intestinal inflammation.

## 1. Introduction

Inflammatory bowel diseases (IBD), including Crohn’s disease and ulcerative colitis (UC), are characterized by chronic relapsing intestinal inflammation. The dysregulation of immune responses is an important pathophysiological mechanism in IBD. Novel biological agents that selectively target specific pathways and molecules have recently been developed for IBD management [1]. Despite the success of these treatments, they may be ineffective in some patients [2]. Systemically delivered immunosuppressants increase the risk of serious infections and other side effects. Therefore, there is a need to explore new mechanism-based targets for IBD treatment.

Sirtuins (SIRTs) are a family of proteins that mainly function as nicotinamide adenine dinucleotide-dependent deacetylases [3]. SIRTs play a role in various physiological processes, including the regulation of metabolism, cell survival, proliferation, and DNA repair [4]. In humans, there are seven sirtuin proteins, SIRT1 to SIRT7 [3]. Among them, SIRT1, a sirtuin of nuclear origin, is associated with the pathogenesis of IBD in various experimental models of colitis [5]. SIRT1 activation enhances the intestinal barrier and reduces the apoptosis of intestinal epithelial cells [6,7]. However, in a human trial, an oral SIRT1 activator did not demonstrate significant clinical activity in patients with active UC [8]. Another sirtuin with a nuclear origin that has recently received attention is sirtuin 7 (SIRT7). SIRT7 is involved in various processes such as DNA damage repair, cell signaling, and aging [9,10,11]. SIRT7 has been found to be involved in various immune-mediated inflammatory responses, such as the NF-kB inflammatory pathway [12]. Therefore, it is possible that SIRT7 is closely related to intestinal immune-mediated inflammatory responses such as IBD [13,14]. Thus far, the exact role of SIRT7 in intestinal inflammation has not been well investigated.

We previously confirmed subsequent inflammatory changes in the large intestine when inflammatory reactions were induced in the lungs of the mice by nasal stimulation of ovalbumin (OVA); we named this the “ovalbumin-induced colitis model” [15]. In our preliminary study, using an OVA-induced colitis model, we found that SIRT7 levels in the lungs were increased when inflammatory responses were induced. Considering the potent effects of SIRT7 and its function in inflammation, we hypothesized that SIRT7 is involved in colitis, especially immune-mediated intestinal inflammation. To this end, we first studied whether colonic inflammation is associated with alterations in colonic SIRT7 expression in mice. We assessed the effect of a novel selective SIRT7 inhibitor on the progression of colitis in our experimental animal model. Furthermore, we measured changes in SIRT1 levels to determine the association between SIRT7 and SIRT1 during intestinal inflammation.

## 2. Materials and Methods

### 2.1. Mouse Immune-Mediated Intestinal Inflammation Model

All procedures were approved by the Institutional Animal Care and Use Committee of Korea University College of Medicine (approval no. KOREA-2020-0073). All experiments were conducted in accordance with the National Institutes of Health Guide for the Care and Use of Laboratory Animals, the ARRIVE guidelines, and other relevant guidelines and regulations. In this study, we used the OVA-induced colitis murine model used in our previous studies [15].

Four-week-old female BALB/c mice (Orient, Daejeon, Korea) were bred in conventional pathogen-free animal facilities. Mice were divided into three groups (*n* = 10 each): control, OVA-induced colitis, and SIRT7 inhibitor (with OVA) treatment. An OVA-induced colitis murine model was established as previously described.

**Figure 1:**
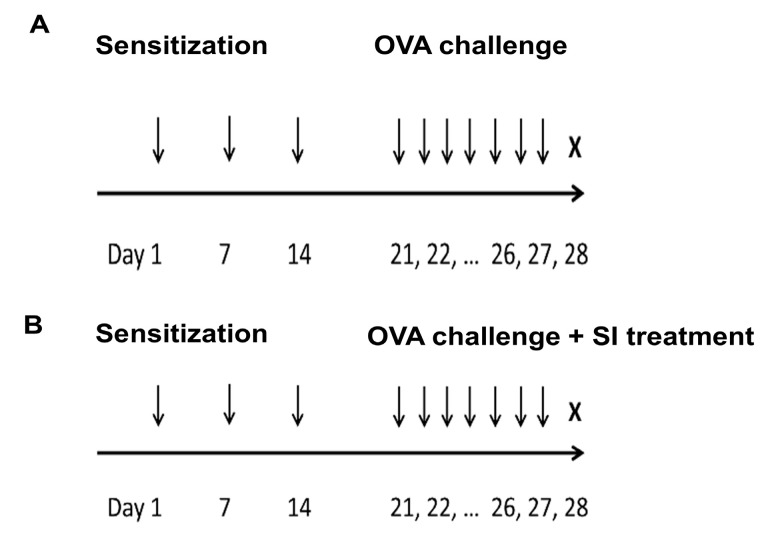
Protocol for the induction of colitis and sirtuin 7 (SIRT7) inhibitor treatment in mice. (**A**) The timeline illustrates that colitis mice were intraperitoneally injected with ovalbumin (OVA) and subsequently intranasally administered OVA from days 21 to 27. Mice were sacrificed on day 28. (**B**) Mice of the SIRT7 inhibitor-treated group were treated with SIRT7 inhibitor (SI). Mice were intranasally challenged with SIRT7 inhibitor and OVA every alternate day from days 21 to 27. Mice were sacrificed on day 28.

Mice were sensitized by intraperitoneal injection of 25 μg of OVA (Sigma-Aldrich, St Louis, MO, USA) suspended in 1% aluminum hydroxide (Thermo Fisher Scientific, Waltham, MA, USA) on days 1, 7, and 14. In the colitis group, on days 21–27, the mice were intranasally challenged with 50 μL of OVA (10 mg/mL) mixed with saline (Figure 1A). The control group was sensitized and challenged with saline instead of OVA. An intranasal challenge with OVA was performed by holding the mouse in a supine position and instilling a drop under the nose. In the SIRT7 inhibitor (SI)-treated group, 100 µM of SIRT7 inhibitor (Korea Research Institute of Chemical Technology, Daejeon, Korea) and OVA were administered intranasally every alternate day on days 21–27 (Figure 1B). The mice were sacrificed, and lung and colon samples were collected.

### 2.2. Immunohistochemical Staining

Paraffin-embedded mouse colon sections were prepared using standard procedures. Immunohistochemical staining was performed in accordance with routine protocols. Tissue sections were incubated with primary antibodies against SIRT7 (Santa Cruz Biotechnology, Dallas, TX, USA) and SIRT1 (Santa Cruz Biotechnology, Dallas, TX, USA) overnight at 4 °C. Sections were washed with PBS and incubated with secondary antibody at room temperature for 1 h. The slides were examined under an Olympus BX51 microscope (Olympus Corporation, Tokyo, Japan) by two independent pathologists. Data were captured using an Olympus DP72 microscope digital camera (Olympus Corporation) with DP2-BSW software.

### 2.3. qRT-PCR for Inflammatory Cytokines and SIRT7 in the Intestinal Mucosa

qRT-PCR was performed on the colonic mucosal tissues. Localized expression of IFN-γ, IL-1β, IL-4, IL-5, IL-13, SIRT1, and SIRT7 levels were evaluated. Total RNA was isolated from 100 mg of colonic tissue using an RNA Extraction Kit (Takara, Maebashi, Japan) and processed with the PrimeScript RT Reagent Kit (Takara) to synthesize cDNA. The mRNA was purified by treatment with chloroform, isopropanol, and ethanol. cDNA was synthesized from RNA using the cDNA Synthesis Master Mix (GenDEPOT, Katy, TX, USA). The cDNA was amplified and quantified using SYBR Green Master Mix (Qiagen, Hilden, Germany). Primer sequences are listed in Table S1 qRT-PCR was performed in triplicate for each sample using a real-time thermal cycler (TP850; Takara). The reaction conditions for DNA amplification were as follows: 95 °C for 120 s, followed by 50 cycles at 95 °C for 15 s and 60 °C for 45 s. Target mRNA expression was normalized to that of glyceraldehyde 3-phosphate dehydrogenase. The data were analyzed using the ΔCt method.

### 2.4. Histological Evaluation of Inflammation

The mice were sacrificed, their colons were removed, and the colon length and weight of each mouse were measured. The stool characteristics inside the colon were also recorded using the stool score index [16]. Hematoxylin and Eosin (H & E) staining was performed on paraffin-embedded, 4 mm thick sections of Swiss roll colon specimens. For histopathological analysis, H & E-stained sections were examined by two independent pathologists in a blinded manner using an Olympus BX51 microscope (Tokyo, Japan). Based on a previously described method, an inflammatory score ranging from 0 to 3 (0 = none, 1 = mild, 2 = moderate, and 3 = severe) was assigned [17]. The inflammatory area (%) was defined as the proportion of the area invaded by inflammatory cells compared to the total mucosal area. The area of inflammatory lesions was calculated in four contiguous, nonoverlapping fields using ImageJ (US National Institutes of Health, Bethesda, MD, USA) software at 200× magnification in a blinded manner by two independent pathologists.

### 2.5. Statistical Analysis

Statistical analyses were conducted using SPSS (version 20.0; IBM Corp., Armonk, NY, USA) and GraphPad Prism software (San Diego, CA, USA). Clinical characteristics and experimental data were compared using Student’s *t*-test (two-tailed). The results are expressed as mean ± standard error of the mean (SEM) or standard deviation (SD). Statistical significance was set at *p* < 0.05. Error bars represent SEM or standard deviation (SD).

## 3. Results

### 3.1. SIRT7 Expression in Lung Tissue Samples from Ovalbumin-Induced Inflammation Mice

**Figure 2:**
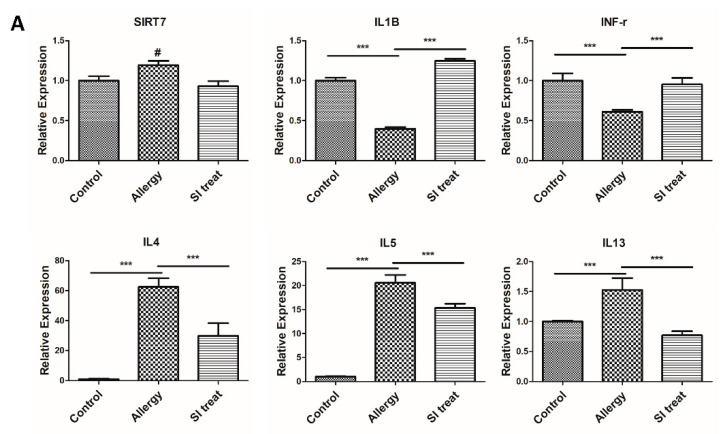

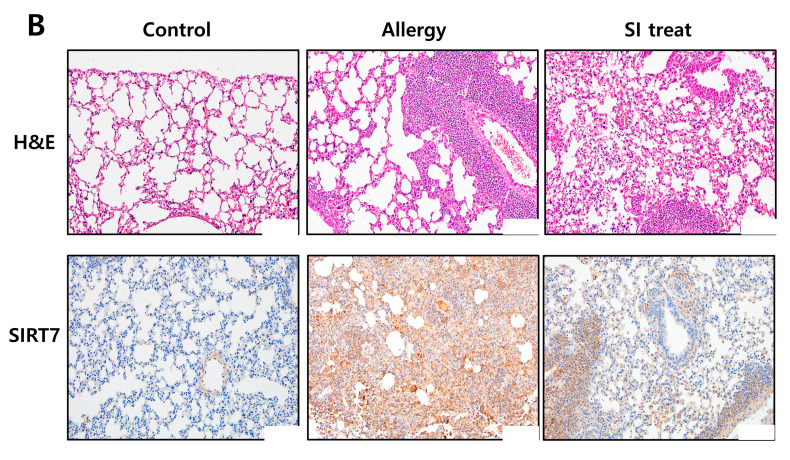
Expression of sirtuin (SIRT)7 and inflammatory cytokines in lung tissues of the ovalbumin (OVA)-induced inflammation murine model. SIRT7 inhibition attenuated OVA-induced allergic inflammation of the lung. (**A**) Comparison of SIRT7 and inflammatory cytokine expression between the control group, allergic inflammation group, and SIRT7 inhibitor (SI)-treated group with qRT-PCR. (**B**) Hematoxylin and eosin staining of lung tissues of the control group, allergic inflammation group, and SI-treated group. Immunohistochemical staining of SIRT7 in lung tissues of the control group, inflammation group, and SI-treated group. Error bars indicate the mean ± standard error of the mean. # *p* = 0.065, *** *p* < 0.05. White scale bar = 50 μm.

In an OVA-induced colitis model, OVA simultaneously induced colitis and allergic inflammation in the lungs. We induced inflammation in the lungs and colons of female BALB/c mice and performed multiple tests to confirm that the OVA-induced lung inflammation/colitis model was successfully established [15]. We decided to analyze SIRT7 in lung allergic inflammation as a preliminary study before colon evaluation. We profiled the messenger RNA (mRNA) expression of SIRT7 in lung tissues of the allergic inflammation and control groups. The results showed that lung tissue samples from the allergy group showed an increasing trend in SIRT7 expression levels compared to those from the control group (*p* = 0.065; Figure 2A). Immunohistochemistry (IHC) staining showed that SIRT7 was significantly upregulated in lung tissue samples from the allergic inflammation mice group compared to that in the control group (Figure 2B).

### 3.2. SIRT7 Inhibitor Attenuated Lung Inflammation by Downregulation of SIRT7

We investigated the expression of inflammatory cytokines and their corresponding mRNAs in the lung tissues of mice with allergic inflammation. qRT-PCR results showed that IL-4, IL-5, and IL-13 levels were significantly higher in the lung tissue of mice with inflammation than in mice in the control group (*p* < 0.05; Figure 2A). H & E staining of lung tissues from allergic mice showed increased immune cell infiltration and goblet cell metaplasia compared to those from control mice (Figure 2B). We then observed changes in lung tissue SIRT7 and inflammatory cytokines when administering SIRT7 inhibitor (SI) to the allergic mouse group. Our data showed that SIRT7 inhibition significantly reduced the mRNA expression of IL-4, IL-5, and IL-13 (*p* < 0.05; Figure 2A). IFN-γ and IL-1β, which were significantly decreased in the allergy group, increased significantly after SI treatment (*p* < 0.05; Figure 2A). Increased SIRT7 levels in the allergic mouse group appeared to decrease on immunohistochemical staining after SI administration (Figure 2B). Mice with inflammation treated with SI showed decreased goblet cell metaplasia and immune cell counts in the lung tissues compared to those of mice not administered SI (Figure 2B).

### 3.3. SIRT7 Expression in Colonic Mucosal Samples from Ovalbumin-Induced Colitis Mice

**Figure 3:**
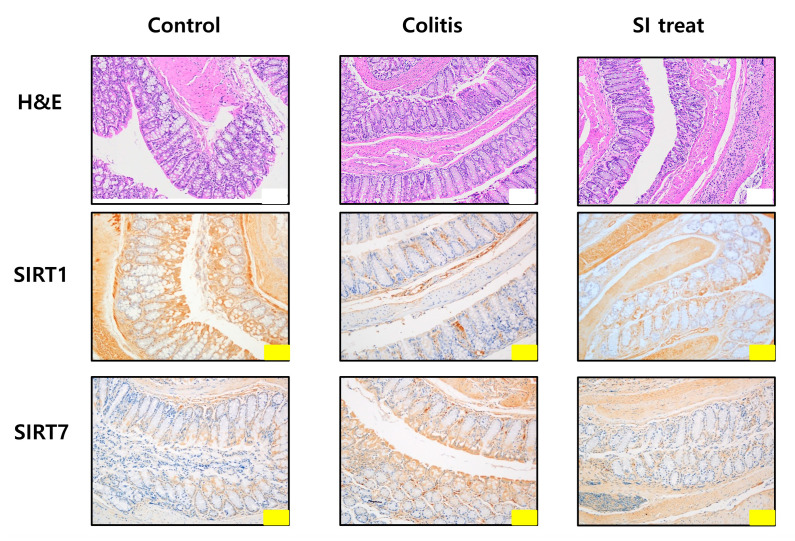
Microscopic analysis of the colonic mucosa in mice. Histological analysis of the colonic mucosa of mice in the control, colitis, and SI-treated groups after hematoxylin and eosin staining. Immunohistochemical staining of SIRT1 and SIRT7 in the colonic mucosa. Yellow scale bar = 100 μm, white scale bar = 200 μm.

**Figure 4:**
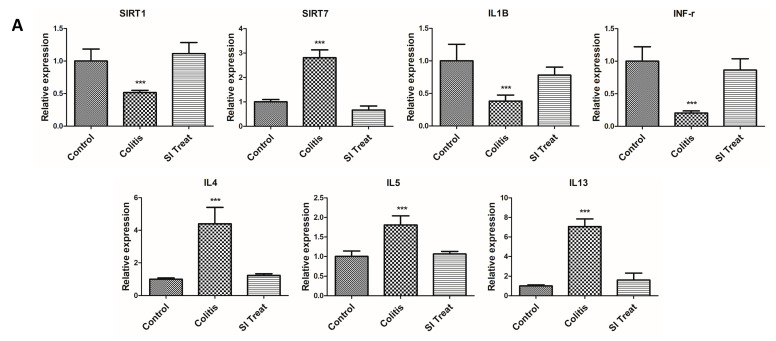

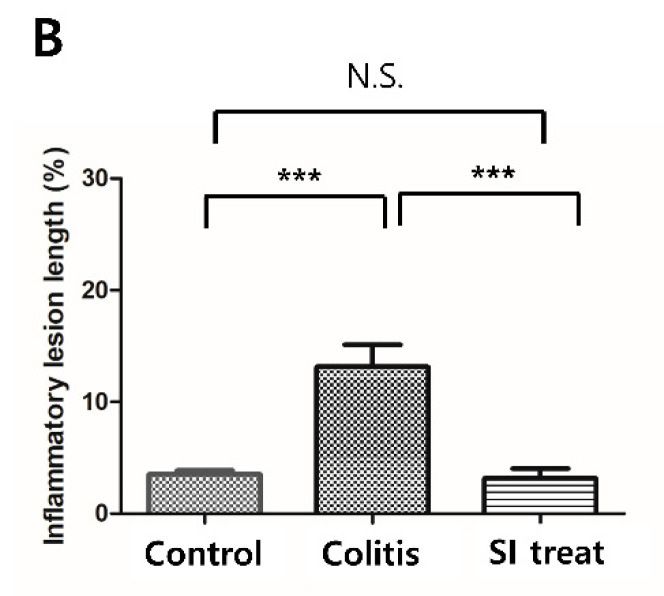
SIRT7 inhibitor reduced colonic inflammation. (**A**) Comparison of SIRT1, SIRT7, and inflammatory cytokine expression in the colon mucosa between the control, colitis, and SIRT7 inhibitor (SI)-treated groups with qRT-PCR. (**B**) The calculated area of the intestinal mucosa with inflammatory cell infiltration was compared to the total mucosal area of the sample. The percentage of the inflammatory area was compared among the groups. *** *p* < 0.05. N.S., not significant.

To evaluate SIRT7 expression and its association with inflammatory responses typical of colitis in vivo, mRNA levels of IFN-γ, IL-1β, IL-4, IL-5, IL-13, SIRT1, and SIRT7 were measured in colon samples from mice. To examine whether colonic inflammation affected SIRT7 expression in the mouse colon, we performed immunohistochemical staining of the colon mucosa of colitis-induced mice. Immunohistochemical staining showed that SIRT7 was significantly upregulated in the colon mucosa samples from colitis-induced mice compared to those of mice in the control group (Figure 3). The qRT-PCR results showed that IL-4, IL-5, and IL-13 levels were significantly higher in the intestinal mucosa of colitis-induced mice than those in the control group (*p* < 0.05; Figure 4A). IFN-γ and IL-1β were significantly reduced in the colitis group compared to those in the controls (*p* < 0.05; Figure 4A).

### 3.4. SIRT7 Inhibitor Decreased Colonic Inflammation by Downregulation of SIRT7

After administration of SI, IL-4, IL-5, and IL-13 levels were significantly reduced in the colonic mucosa of SI-treated mice compared to those in the colitis group (*p* < 0.05; Figure 4A). IFN-γ and IL-1β levels were significantly higher in the intestinal mucosa of SI-treated mice than in the mice of the colitis group (*p* < 0.05; Figure 4A). Immunohistochemical staining showed that SIRT7 was significantly downregulated in colon mucosa samples from SI-treated mice compared to those of mice in the colitis group (Figure 3).

### 3.5. Changes in SIRT1 Level in the Colonic Mucosa of Colitis Mice

We evaluated changes in SIRT1 levels in the colon of the control, colitis, and SI-treated groups using qRT-PCR. SIRT1 expression in the murine colonic mucosa of mice in the colitis group was lower than that of mice in the control group (Figure 4A). SIRT1 levels were significantly higher in the SI-treated group than those of mice in the colitis group. Immunohistochemical staining showed that SIRT1 was significantly upregulated in colon mucosa samples from SI-treated mice compared to those of mice in the colitis group (Figure 3).

### 3.6. Histopathological Analysis of the Colonic Mucosa

Inflammation and epithelial cell degeneration were assessed in H&E-stained colon sections of mice from the control, colitis, and SI-treated groups. No gross morphological differences, such as inflammatory infiltration by lymphocytes, epithelial erosion, or crypt loss, were observed among the mice in the three groups (Figure 3). Therefore, the sum of the proportions of the inflammatory lesions in each group was compared. A significant increase in the percentage of the inflammatory area was observed in colitis-induced mice (control group: 3.51 ± 0.33%; colitis group: 13.14 ± 1.9%; *p* < 0.05) (Figure 4B). Compared with the colitis group, the SI-treated group showed a significant decrease in the extent of inflammatory lesions (colitis group: 13.14 ± 1.9%; SI-treated group: 3.17 ± 0.86%; *p* < 0.05).

### 3.7. Macroscopic Scoring of Colon Changes

Macroscopic changes in the colon were evaluated by measuring body weight, colon length, and stool characteristics of colitis and SI-treated mice. The stool score index of the severity of colonic changes described by Kimball et al. [16] was used. Colon length was shorter in the colitis group than in the control group (*p* < 0.05; Table 1). The colon length of mice in the SI-treated group did not show a significant change compared to that of mice in the control group. Mice with colitis had bright brown-colored watery stools, whereas mice in the control and SI-treated groups had dark-brown solid feces. There were no significant differences in colon weight or stool score between the mice in the control and the SI-treated groups (Table 1).

## 4. Discussion

In this study, we successfully evaluated a novel pathway that mediates the immune-inflammatory response of the colon and a new drug that targets it in a murine model of colitis. The major findings of this report are as follows: (1) acute inflammation in the colonic mucosa is associated with increased SIRT7 and decreased SIRT1 levels; (2) inhibition of SIRT7 reduces the expression of inflammatory cytokines; and (3) measurements of the length, weight, and stool characteristics of the mice confirms that treatment with SIRT7 inhibitors effectively improves colitis.

Primarily, this study is meaningful in that it is the first to investigate the role of SIRT7 in colon immunity. Previous studies have shown that SIRT1 plays a role in regulating inflammatory signals in the gastrointestinal tract. Studies have demonstrated an inhibitory effect of SIRT1 on nuclear factor kappa B (NF-κB)-mediated inflammation of the gastrointestinal tract. Activation of SIRT1 or overexpression of SIRT1 by resveratrol promotes deacetylation of p65 and suppression of transcriptional activation by NF-kB [18]. However, in a human trial, an oral SIRT1 selective activator did not demonstrate significant clinical activity in mild to moderately active UC [8]. Although the enzymatic activities of SIRT7 have not been well characterized, recent studies have revealed that SIRT7 exhibits deacetylase, desuccinylase, and deacylase activities and is critical for responses to damaged DNA, tumorigenesis, tissue repair, regulation of ribosomal RNA transcription, and mitochondrial function [19,20,21]. Recent studies have focused on the role of SIRT7 in inflammation. SIRT7 has been implicated in the inflammation of the kidneys, heart, and lungs [22,23]. Whether SIRT7 is involved in the induction and progression of intestinal inflammatory response remains unknown. We speculated that SIRT7 may play a role in gastrointestinal inflammation for several reasons. First, SIRT7 antagonizes SIRT1, a key sirtuin involved in colon inflammation. Studies have also found that a lack of SIRT7 suppresses the nuclear accumulation of p65, a component of NF-κB. Fang et al. [24] described the mechanism by which SIRT1 activity is regulated by SIRT7. SIRT1 can increase its autocatalytic activity via autodeacetylation [25]. Studies have shown that SIRT7 binds to SIRT1 and inhibits autodeacetylation, thereby suppressing its activity [26]. Second, SIRT7 regulates the nuclear export of NF-κB. The lack of SIRT7 suppresses the nuclear accumulation of p65, which is a component of NF-κB [27].

**Figure 5:**
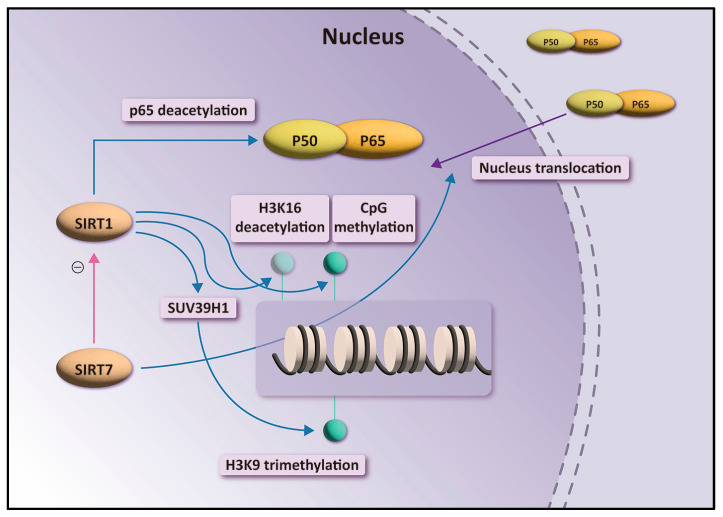
Overview of the role of SIRT1 and SIRT7 in the transactivation of NF-κB-dependent gene expression.

NF-κB is an important transcription factor involved in inflammatory response, cell proliferation, and apoptosis in cells. NF-κB comprises p50 and p65 (RelA) subunits, which associate with the inhibitory factor IκB in the cytoplasm of unstimulated cells [28]. NF-κB is activated in response to various stimuli, leading to the degradation and release of IκBα from the dimeric complex. Phosphorylation of NF-κBp65 and its subsequent translocation to the nucleus occurs [29]. When NF-κBp-p65 enters the nucleus, NF-κB initiates the transcription of NF-κB-dependent proinflammatory genes [30]. SIRTs regulate the NF-κB pathway in various ways [31]. SIRT1 interacts with NF-κBp65 to inhibit NF-κB-associated transcription and suppresses inflammation in multiple tissues and macrophages [32]. SIRT1 suppresses transcription of inflammatory genes by enhancing the activities of histone methyltransferases [33]. SIRT1 deacetylates and activates the histone methyltransferase SUV39H1, resulting in increased levels of trimethyl H3K9, which inhibits the expression of inflammatory genes [34,35]. SIRT1 represses inflammatory responses by deacetylating H4K16 and recruiting nonhistone proteins, such as p65/RelA [36]. Recent studies have demonstrated that SIRT7 interacts with SIRT1. SIRT7 possibly functions through SIRT1 in NF-κB acetylation (Figure 5) [24]. Chen [31] revealed that SIRT7 participates in the nucleocytoplasmic translocation of NF-κBpp65 and NF-κB phosphorylation. As SIRT7 is mainly concentrated in the nucleus, SIRT7 binds to p65 in the nucleus and regulates the translocation of p65 between the nucleus and cytoplasm (Figure 5) [27].

In our preliminary study, we confirmed that SIRT7 was elevated in the lungs of mice that induced allergic inflammation of the lung and hypothesized that a similar response would also occur in the colon mucosa that induced colitis [15]. We further evaluated whether SIRT7 inhibitors attenuated inflammation in a colitis model. We hypothesized that the mechanism by which SIRT 7 acts on intestinal inflammation is related to the NF-kB pathway and investigated whether SIRT1 levels also change with SIRT7 inhibition.

The results of the present study suggest that SIRT7 inhibition suppressed the OVA-induced inflammation of the colonic mucosa. The results of the qRT-PCR analysis of colonic tissues of colitis mice showed changes in the expression of inflammatory cytokines after SI treatment. Because our colitis model was induced through an allergic response, Th2 cytokines increased and Th1 cytokines decreased after colitis induction. In our experiment, the altered inflammatory cytokines were normalized after SI treatment. We observed a significant decrease in the relative length of inflammatory lesions in the colonic mucosa of mice with colitis after SI treatment. We also found that the degree of clinical inflammation was improved in SI-treated mice. Notably, in our study, SIRT1 levels changed antagonistically with SIRT7. We assume that elevated SIRT7 levels inhibit SIRT1 activity.

In this experiment, we used the OVA-induced inflammation murine model used in our previous study [15]. Briefly, it is a model wherein OVA simultaneously induces inflammation in the lungs and intestines through immune migration. Administration of OVA nasally initiates colonic inflammation, leading to weight loss, watery diarrhea, and histopathological features that mimic clinical conditions of chronic relapsing intestinal immune-mediated inflammation, such as IBD or irritable bowel syndrome. Among the various animal experimental colitis models, this model emphasizes the immunological aspects of colon inflammation.

Our study had several limitations. First, we did not evaluate the detailed molecular mechanisms of SIRT7 or the NF-κB pathway in colitis. In the present study, we provide experimental evidence that SIRT7 may act as a regulator of immune-mediated intestinal inflammation via the NF-κB pathway. Further investigation is needed to solve the overall complexity of the regulatory networks that fine-tune the activities of various SIRTs. Second, our animal model may have limitations in representing IBD such as UC. In our experimental group, no findings suggestive of IBD, such as frequent diarrhea or mucus/bloody stool, were observed. Animal models for IBD experiments usually use 3.5% sodium dextran sulfate (DSS) to induce acute colitis [28]. However, instead of inducing colitis through direct injury from a chemical agent, we induced an intestinal inflammatory response through systemic immune activation, as in our previous study [15].

In summary, our findings indicate that SIRT7 attenuates colonic mucosal inflammation in mice. Because NF-κB plays an important role in many inflammatory and metabolic diseases, there is considerable interest in whether the inhibition of SIRT7 is useful for treating immune-mediated intestinal inflammation. SIRT1 activators have been shown to suppress NF-κB-induced transcription and inhibit proinflammatory cytokine secretion in vitro [37,38]. However, in a human trial, an oral SIRT1 activator did not demonstrate significant clinical activity in patients with active IBD [8]. Therefore, suppressing SIRT7 may represent a new approach for attenuating NF-κB activity in immune-mediated intestinal inflammatory diseases such as IBD.

## 5. Conclusions

In conclusion, our study shows that SIRT7 may play a role in the inflammatory response in the intestine. Our study demonstrated that SIRT7 inhibition blocks the inflammatory response in the colon of mice, suggesting a possible role for SIRT7 in the pathogenesis of immune-mediated intestinal inflammation.

## Figures and Tables

**Table 1:** Colonic changes including the measured colon length, colon weight, and stool score were analyzed as described in the Materials and Methods. Data are presented as mean ± standard deviation (n = 10 per group, * *p* < 0.05 vs. control, ANOVA).

Parameter	Control	Colitis	SI-Treated
Colon length, cm	10.48 ± 0.19	9.06 ± 0.20 *	10.22 ± 0.29
Colon weight, g	0.486 ± 0.014	0.494 ± 0.023	0.476 ± 0.031
Stool score	0.3 ± 0.14	1.1 ± 0.16 *	0.5 ± 0.17

## Data Availability

The datasets used in this study are available from the corresponding author upon reasonable request.

## Supplementary Materials

The following are available online at https://www.mdpi.com/article/10.3390/biomedicines10112693/s1: Table S1: Primer and probe sequences for murine chemokines and other immune-related factors.
