# Supplementary material for: Sirtuin 7 Inhibitor Attenuates Colonic Mucosal Immune Activation in Mice—Potential Therapeutic Target in Inflammatory Bowel Disease
Source: Biomedicines. 2022 Oct 25;10(11):2693. doi: 10.3390/biomedicines10112693 (PMC9687268; doi:10.3390/biomedicines10112693)
Supplement: Supplementary file 1 [file biomedicines-10-02693-s001.zip › biomedicines-1934790-supplementary.pdf]

Supplementary material

Table S1. Primer and probe sequences for murine chemokines and other immune-related factors.

Gene name	Forward primer (5'–3')	Reverse primer (5'–3')
Mouse GAPDH	TTCAACAGCAACTCCCACTC	TCCTTGGAGGCCATGTAGG
Mouse IFN-γ	TCAAGTGGCATAGATGTGGAAGAA	TGGCTCTGCAGGATTTTCATG
Mouse SIRT1	CGGCTACCGAGGTCCATATAC	CAGCTCAGGTGGAGGAATTGT
Mouse SIRT7	TGCCAGGCACTTGGTTGTCT	TAGGCTCCGCTTCGCTTAGG
Mouse IL-1b	CCTTCCAGGATGAGGACATGA	TGAGTCACAGAGGATGGGCTC
Mouse IL-4	ACAGGAGAAGGGACGCCAT	GAAGCCCTACAGACGAGCTCA
Mouse IL-5	AGGCTTCCTGTCCCTACTCAT	TACCCCCACGGACAGTTTG
Mouse IL-13	TCAAGTGGCATAGATGTGGAAGAA	TGGCTCTGCAGGATTTTCATG

GADPH, glyceraldehyde-3-phosphate dehydrogenase; IFN, interferon; SIRT, sirtuin; IL, interleukin
